# Evaluation of shorter versus longer antifungal treatment durations for *Candida spp*. urinary tract infections among hospitalized adults

**DOI:** 10.1128/aac.01920-24

**Published:** 2025-04-22

**Authors:** Jacob C. Govel, Robert W. Seabury, Elizabeth A. Asiago-Reddy, Ramiro L. Gutierrez, Katie A. Parsels, Wesley D. Kufel

**Affiliations:** 1 State University of New York at Binghamton School of Pharmacy and Pharmaceutical Sciences 465824, Binghamton, New York, USA; 2 State University of New York Upstate University Hospital, Syracuse, New York, USA; 3 State University of New York Upstate Medical University, Syracuse, New York, USA; Johns Hopkins University School of Medicine, Baltimore, Maryland, USA

**Keywords:** *Candida*, fluconazole, urinary tract infection, duration, antifungal

## Abstract

Infectious Diseases Society of America guidelines recommend 14 days of treatment for *Candida spp*. urinary tract infections (UTIs). To our knowledge, no data are available to compare <14 days for *Candida spp*. UTI. This was a single-center, retrospective cohort study between 01 January 2015 and 01 January 2024. Hospitalized adults with >1 urine culture with *Candida spp*. and symptoms who initiated >1 antifungal dose within 96 hours were included. Multiple exclusion criteria existed, including but not limited to if *Candida spp*. were isolated from another site, antifungals were received for another indication, or the participant was asymptomatic. The primary outcome was clinical treatment success. Binary logistic regression was performed to further assess the relationship between fluconazole duration and clinical treatment success. Among 2,400 patients with candiduria, 45 and 58 in the 14-day and <14-day cohorts were assessed after exclusion criteria were applied, respectively. Median (interquartile range) fluconazole duration was 14 (14–14) days in the 14-day cohort and 7 (5–7) in the <14-day cohort. There was no difference in clinical treatment success in patients treated for 14 days vs <14 days (14 days: 93.3% (42/45) vs <14 days: 93.1% (54/58), *P* = 1.000; between-group difference (95% CI: 0.02 [−9.6 to 10]). Fluconazole duration did not have a significant association with clinical treatment success on binary logistic regression (*P* = 0.503; odds ratio 0.917 [95% CI: 0.712–1.181]). There was no statistically significant difference in clinical treatment success in patients treated with fluconazole for a median of 14 days vs a median of 7 days for symptomatic *Candida spp*. UTI. These data support the potential utility of shorter antifungal durations for *Candida spp*. UTI.

## INTRODUCTION

Candiduria is common among hospitalized patients (1, 2). Those who are most at risk include patients who are critically ill, have urinary tract devices *in situ* (e.g., indwelling foley catheters, nephrostomy tubes, and urinary stents), have complex urinary anatomy and/or urinary surgical procedures, received prior antibiotic therapy, and have diabetes mellitus and/or malignancy (3–5). Most patients with candiduria are asymptomatic and are colonized with *Candida spp*. in the urinary tract (3). Elimination of underlying risk factors, such as the removal of indwelling catheters, is typically adequate to eradicate candiduria (3). Despite these interventions, candiduria can still progress to symptomatic urinary tract infections (UTI) (3, 6). These patients will generally present with cystitis or pyelonephritis symptoms similar to bacterial UTI (e.g., dysuria, polyuria, urinary urgency, costovertebral angle [CVA] tenderness, and/or fever).

The 2016 Infectious Diseases Society of America (IDSA) guidelines for the management of candidiasis recommend 14 days of antifungal treatment for symptomatic *Candida spp*. UTI (6, 7). However, this recommendation is based on low-quality evidence (6, 7). For bacterial UTI, there are ample data to support using shorter antibiotic durations (e.g., 7 days) compared to longer durations (e.g., 14 days) even in cases of associated gram-negative bacteremia (8–11). However, data are lacking to compare shorter vs longer antifungal treatment durations for symptomatic *Candida spp*. UTI. Therefore, we sought to evaluate shorter antifungal treatment durations compared to 14 days.

## MATERIALS AND METHODS

### Study outcomes

The primary outcome was to evaluate clinical treatment success among shorter vs longer antifungal treatment durations for symptomatic *Candida spp*. UTI. Secondary outcomes included hospital readmission within 90 days of index culture, hospital readmission within 90 days of index culture due to *Candida spp*. UTI, microbiological recurrence within 90 days of index culture, and all-cause mortality within 90 days of index culture.

### Study design

This was a single-center, retrospective cohort study between 01 January 2015 and 01 January 2024 at the State University of New York (SUNY) Upstate University Hospital, a 752-bed, tertiary care, academic medical center in Syracuse, New York. Our institution is recognized as an Antimicrobial Stewardship Center of Excellence by IDSA. Patients 18 years of age or older with at least one urine culture and over 10,000 colony-forming units per milliliter of *Candida spp*. along with symptoms of UTI, including dysuria, polyuria, urinary urgency, hematuria, CVA tenderness, and/or fever who received more than one antifungal dose within 96 hours of index urine culture, were eligible for inclusion. Patients were excluded if they had *Candida spp*. isolated from another culture site (e.g., candidemia and intra-abdominal infection); had received antifungal treatment within 7 days of the index culture; had fluconazole non-susceptible *Candida spp*. (i.e., *Candida krusei* or fluconazole non-susceptible dose-dependent *Candida glabrata*); had a diagnosis of vulvovaginal candidiasis; had a fluconazole allergy; initiated antifungal treatment >96 hours following the index culture; had no symptoms identified but received antifungal treatment (i.e., asymptomatic candiduria); had concurrent bacteriuria or a polymicrobial urine culture; had an outpatient urine culture with no inpatient admission; had an antifungal given empirically for another potential fungal infection; had a duplicate encounter; or were pregnant incarcerated. This study was deemed exempt by the Institutional Review Board at SUNY Upstate Medical University.

### Data collection

Microbiologic reports with urine cultures positive for *Candida spp*. between 01 January 2015 and 01 January 2024 were queried. Inclusion and exclusion criteria were applied. Data were then collected from the electronic medical record (Epic) using a data collection tool developed in Research Electronic Data Capture (REDCap) at SUNY Upstate Medical University.

Patient demographics and comorbidities, including leukemia or lymphoma, solid tumor malignancy, renal cell carcinoma, urologic malignancy, diabetes mellitus, chronic kidney disease stage 3 or higher, and moderate-severe liver disease, were collected. Baseline characteristics, including intensive care unit (ICU) admission, admission location, receipt of antibiotics within 96 hours of a urine culture growing *Candida spp*., immunosuppressive medications, urologic procedures, presence of a urinary catheter, nephrostomy tube, ureteral stent, ileal conduit, or nephrolithiasis at time of index culture, and serum creatinine, were also collected.

Symptoms, including capacity to endorse urinary symptoms, dysuria and/or suprapubic pain, polyuria, increased urinary urgency, CVA tenderness, hematuria, and fever (>38°C), were collected. Fever was assessed for pyelonephritis as well as for patients who were unable to endorse urinary symptoms but had a fever without another attributable infectious process at the clinical judgment of the infectious disease (ID) physician. These infection characteristics were collected via the review of systems and/or physical examination sections of the progress notes within 24 hours of the index culture. Imaging, including computed tomography and/or renal ultrasounds, was also evaluated if obtained within 24 hours of the index culture, and the impression was reviewed to determine if there were supportive findings for UTI. The *Candida spp*. for the symptomatic UTI was also collected from the positive urine culture. Antifungal susceptibility testing is not routinely performed on urine culture isolates unless requested at our institution. Other treatment characteristics collected included receipt of vasopressors, mechanical ventilation, urology consultation, ID consultation, fluconazole dose, and fluconazole treatment duration. Patients were divided based on antifungal treatment duration into “14 days” or “<14 days” cohorts for comparison.

Fluconazole requires ID physician approval for continuation beyond 24 hours (i.e., after one dose) as part of our antimicrobial stewardship program. If patients received only one dose of fluconazole, they were excluded as mentioned previously. Therefore, all patients who received fluconazole for *Candida spp*. UTI were reviewed by an ID physician and supported fluconazole treatment based on their review and clinical judgment of the patient.

### Definitions

Pyelonephritis/febrile UTI was defined as fever with or without costovertebral angle tenderness. Clinical treatment success was defined as any UTI symptom resolution and/or improvement based on progress note documentation, no extension of antifungal treatment duration for longer than originally prescribed, no new additional antifungal prescription within 14 days of treatment completion (including outpatient prescriptions from our institution), and no change in antifungal agent. Treatment duration represented fluconazole received in both inpatient and outpatient post-discharge settings, if applicable. Hospital readmission within 90 days of index culture due to *Candida spp*. UTI was defined as a recurrence of urinary symptoms requiring retreatment with an antifungal. Microbiological recurrence was defined as a urine culture positive for *Candida spp*. within 90 days of index culture.

### Statistical analysis

Statistical analyses were performed using SPSS Version 26.0 (IBM Corp., Armonk, NY) and Excel 365 (Microsoft Corp., Redmond, WA). Data were presented using descriptive statistics, including number (*n*) with percentage (%) and median with interquartile range (IQR). Categorical data were compared using *χ*^2^ or the Fisher exact test. Continuous data were compared using the Mann-Whitney *U* test. Furthermore, the between-group difference with 95% CI was estimated for clinical outcomes. Between-group difference was estimated using the equation *p*_1_ – *p*_2_. The 95% CI for the between-group difference was estimated using the equation *p*_1_ – *p*_2_ ± 1.96× SE.

Binary logistic regression was performed to further assess the relationship between fluconazole treatment duration and clinical treatment success (i.e., primary outcome). Univariate analyses were used to compare patients with and without clinical treatment success. Continuous data were compared using the Mann-Whitney *U* test, and categorical data were compared using *χ*^2^ or Fisher exact test. Clinical treatment success was the dependent variable in the binary logistic regression, and fluconazole treatment duration was included as an independent variable, as it was the primary interest variable in this analysis. Other independent variables were considered for inclusion if they did not have missing data and if they had a *P*-value < 0.25 on the univariate analysis comparing patients with and without treatment success.

The association between independent variables being considered for inclusion in the binary logistic regression was assessed using *χ*^2^ or Fisher exact test with Cramer’s *V* for categorical variables and Spearman’s rank correlation coefficient for continuous variables. Independent variables with a *P*-value < 0.05 and either a Spearman’s rank correlation coefficient >0.6 or Cramer’s *V* > 0.25 were considered very strongly associated. In this circumstance, the independent variable with the lower *P*-value on univariate analysis comparing patients with and without clinical treatment success was retained, while the variable with the higher *P*-value was removed. The selected independent variables were then simultaneously entered into the binary logistic regression using an enter model. Goodness-of-fit was assessed using the Hosmer-Lemeshow test, the Omnibus test, and Nagelkerke’s *R* squared. *P*-values, odds ratios (ORs), and 95% CIs were calculated in the binary logistic regression.

All statistical tests were two-tailed, and a *P* < 0.05 was considered statistically significant. The between-group difference for clinical outcomes was considered statistically significant if zero was not contained in the 95% CI for the between-group difference. In the logistic regression, a result was considered statistically significant if the OR 95% CI did not cross one.

## RESULTS

**Fig 1:**
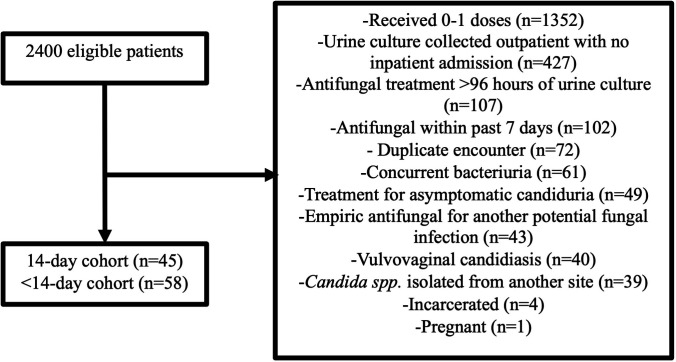
Patient inclusion and exclusion.

Between 01 January 2015 and 01 January 2024, 2,400 patients had a urine culture positive for *Candida spp*. and were eligible for inclusion. After exclusion criteria were applied, 103 patients were included for analysis, with 45 patients and 58 patients in the 14-day and <14-day cohort, respectively. The most common reason for exclusion was receiving 0–1 doses of antifungals (*n* = 1,352). Additional details regarding patients that were excluded are displayed in Fig. 1.

Baseline patient demographics are shown in Table 1. There were no statistically significant differences in demographics, comorbidities, and baseline characteristics between the 14-day cohort and <14-day cohort, suggesting that the two cohorts were generally similar overall. There were numerically more females in the <14-day cohort (62.1%) compared to the 14-day cohort (48.9%). The median age was 67 and 69.5 years in the 14-day and <14-day cohort, respectively. The most common comorbidity was diabetes mellitus with 42.2% in the 14-day cohort and 37.9% in the <14-day cohort. Admission location in the ICU was relatively uncommon with 17.8% and 31% in the 14-day and <14-day cohorts, respectively. Most patients also received concurrent antibiotics for non-UTI indications within 96 hours of the urine culture growing *Candida spp*., with 88.9% in the 14-day cohort and 94.8% in the <14-day cohort. Urologic procedures and urinary catheter/instrumentation data are also presented in Table 1, but there were no statistically significant differences between the 14-day and <14-day cohorts.

**TABLE 1:** Baseline demographics and patient characteristics^*a*^

	14 days (*n* = 45)	<14 days (*n* = 58)	*P*-value
Demographics			
Age (years), median (IQR)	67 (56–77)	69.5 (63–75)	0.692
Female gender, *n* (%)	22 (48.9)	36 (62.1)	0.255
Weight (kg), median (IQR)	75.6 (64.5–90)	78.3 (66.9–92.9)	0.637
BMI (kg/m^2^), median (IQR)	27.3 (23.4–32.2)	27.8 (24.1–33.1)	0.553
Pertinent comorbidities			
Leukemia or lymphoma, *n* (%)	0 (0)	1 (1.7)	1.000
Solid tumor malignancy, *n* (%)	12 (26.7)	8 (13.8)	0.165
Renal cell carcinoma	10 (22.2)	4 (6.9)	0.050
Diabetes mellitus, *n* (%)	19 (42.2)	22 (37.9)	0.812
Chronic kidney disease (>stage 3), *n* (%)	12 (26.7)	9 (15.5)	0.252
Moderate-severe liver disease, *n* (%)	0 (0)	2 (3.4)	0.503
No comorbidities, *n* (%)	14 (31.1)	23 (39.7)	0.491
Baseline characteristics			
ICU admission, *n* (%)	8 (17.8)	18 (31)	0.191
Vasopressors, *n* (%)	5 (11.1)	11 (19)	0.414
Mechanical ventilation, *n* (%)	5 (11.1)	10 (17.2)	0.553
Serum creatinine (mg/dL), median (IQR)	1.17 (0.6–2.0)	1.02 (0.7–1.9)	0.889
Receipt of antibiotics within 96 hours of urine culture with *Candida spp.*, *n* (%)	40 (88.9)	55 (94.8)	0.292
Immunosuppressive medications, *n* (%)	1 (2.2)	2 (3.4)	1.000
Urologic procedure performed, *n* (%)	19 (42.2)	14 (24.1)	0.082
Urinary catheter present at the time of culture, *n* (%)	20 (44.4)	37 (63.8)	0.079
Urinary catheter removal, *n* (%)	17 (85)	35 (94.6)	0.332
Urinary catheter replacement, *n* (%)	10 (50)	19 (51.4)	0.991
No urinary devices *in situ*, *n* (%)	22 (48.9)	40 (69)	0.063
Nephrostomy tube *in situ*, *n* (%)	17 (37.8)	11 (19)	0.057
Ureteral stent *in situ*, *n* (%)	11 (24.4)	9 (15.5)	0.376
Ileal conduit, *n* (%)	2 (4.4)	5 (8.6)	0.464
Nephrolithiasis present, *n* (%)	11 (24.4)	14 (24.1)	1.000

^*a*^ BMI, body mass index.

Symptoms, diagnostics, and treatment characteristics are shown in Table 2. There was a statistically significantly lower number of patients with the capacity to endorse urinary symptoms in the <14-day cohort compared to the 14-day cohort (60.3% vs 82.2%, *P* = 0.029). Most patients experienced more than one symptom and/or fever. In the 14-day cohort, the most common symptoms were fever (46.7%), CVA tenderness (43.2%), and dysuria/suprapubic pain (35.1%). In the <14-day cohort, the most common symptoms were dysuria/suprapubic pain (45.7%), fever (43.1%), and polyuria (25.7%). Pyelonephritis/febrile UTI was more common than cystitis with 82.2% and 70.7% in the 14-day and <14-day cohorts, respectively, but no statistically significant difference was identified. Most patients had imaging supporting UTI in both the 14-day cohort (77.1%) and the <14-day cohort (58.1%). *Candida albicans* was the most common yeast-causing UTI in the 14-day cohort (80%) and the <14-day cohort (62.1%). Receipt of vasopressors and mechanical ventilation was relatively uncommon among both cohorts, and there were no statistically significant differences. Most patients received ID consultation in the 14-day and <14-day cohorts (60% and 62.1%, respectively); however, there was a statistically significant difference in urology consultation with 62.2% and 36.2% (*P* = 0.015) in the 14-day and <14-day cohorts, respectively. The median fluconazole dose in both cohorts was 200 mg, and the use of a loading dose was relatively uncommon. The median (IQR) fluconazole duration was 14 days (14–14) and 7 (5–7) in the 14-day and <14-day cohorts, respectively (*P* < 0.001).

**TABLE 2:** Symptoms, diagnostics, and treatment characteristics^*a*^

	14 days (*n* = 45)	<14 days (*n* = 58)	*P*-value
Symptoms and clinical presentation			
Capacity to endorse urinary symptoms, *n* (%)	37 (82.2)	35 (60.3)	0.029
Dysuria or suprapubic pain, *n* (%)	13 (35.1)	16 (45.7)	0.500
Polyuria, *n* (%)	4 (10.8)	9 (25.7)	0.181
Increased urinary urgency, *n* (%)	4 (10.8)	7 (20)	0.450
CVA tenderness, *n* (%)	16 (43.2)	6 (17.1)	0.032
Hematuria, *n* (%)	7 (18.9)	3 (8.6)	0.309
Fever, *n* (%)	29 (64.4)	36 (60.3)	0.718
Pyelonephritis/febrile UTI, *n* (%)	37 (82.2)	41 (70.7)	0.262
Imaging			
CT A/P and/or renal ultrasound, *n* (%)	35 (77.8)	43 (74.1)	0.845
Imaging supporting UTI, *n* (%)	27 (77.1)	25 (58.1)	0.126
*Candida spp*.			
*C. albicans*, *n* (%)	36 (80)	36 (62.1)	0.080
*C. glabrata*, *n* (%)	2 (4.4)	6 (10.3)	0.461
*Candida parapsilosis*, *n* (%)	2 (4.4)	3 (5.2)	1.000
*Candida lusitaniae*, *n* (%)	0 (0)	3 (5.2)	0.255
*Candida tropicalis*, *n* (%)	5 (11.1)	7 (12.1)	1.000
*Candida kefyr*, *n* (%)	0 (0)	3 (5.2)	0.255
Treatment characteristics			
Urology consultation, *n* (%)	28 (62.2)	21 (36.2)	0.015
ID consultation, *n* (%)	27 (60)	36 (62.1)	0.992
Fluconazole dose (mg), median (IQR)	200 (200–200)	200 (200–200)	0.412
Fluconazole loading dose (mg), *n* (%)	9 (20)	13 (22.4)	0.957
Fluconazole duration (days), median (IQR)	14 (14–14)	7 (5–7)	<0.001

^*a*^ CT A/P, computed tomography of abdomen and pelvis.

Clinical outcomes are displayed in Table 3. For the primary outcome, there was no statistically significant difference in clinical treatment success in the 14-day cohort compared to the <14-day cohort (93.3% vs 93.1%, *P* = 1.000; between-group difference [95% CI]: 0.02 [−9.6% to 10%]). For the secondary outcomes, there were no statistically significant differences between the 14-day cohort and the <14-day cohort for hospital readmission within 90 days of index culture (51.1% vs 37.9%, *P* = 0.255; between-group difference [95% CI]: 13.2 [−6% to 32.4%]), hospital readmission due to *Candida spp*. UTI within 90 days of index culture (11.1% vs 6.9%, *P* = 0.499; between-group difference [95% CI]: 4.2 [−7% to 15.4%]), microbiologic recurrence within 90 days of index culture (15.6% vs 13.8%, *P* = 1.000; between-group difference [95% CI]: 1.8 [−12.1% to 15.6%]), or all-cause mortality within 90 days of index culture (13.3% vs 17.2%, *P* = 0.788; between-group difference [95% CI]: −3.9 [−17.8% to 10%]).

**TABLE 3:** Clinical study outcomes

	14 days (*n* = 45)	<14 days (*n* = 58)	*P*-value	Between cohort difference (95% CI)
Primary outcome				
Clinical treatment success, *n* (%)	42 (93.3)	54 (93.1)	1.000	0.02 (−9.6 to 10)
Secondary outcomes				
Hospital readmission^*a*^, *n* (%)	23 (51.1)	22 (37.9)	0.255	13.2 (−6 to 32.4)
Hospital readmission due to *Candida spp*. UTI^*a*^, *n* (%)	5 (11.1)	4 (6.9)	0.499	4.2 (−7 to 15.4)
Microbiologic recurrence^*a*^, *n* (%)	7 (15.6)	8 (13.8)	1.000	1.8 (−12.1 to 15.6)
All-cause mortality^*a*^, *n* (%)	6 (13.3)	10 (17.2)	0.788	−3.9 (−17.8 to 10)

^*a*^ Within 90 days of index culture.

The following variables had a *P* < 0.25 on the univariate analysis comparing patients with and without clinical treatment success and were considered for inclusion in the binary logistic regression with fluconazole treatment duration (*P* = 0.772): weight (*P* = 0.142), ICU admission (*P* = 0.066), serum creatinine (*P* = 0.146), capacity to endorse urinary symptoms (*P* = 0.194), *Candida tropicalis* UTI (*P* = 0.188), vasopressors (*P* = 0.011), fluconazole loading dose (*P* = 0.165), renal cell carcinoma (*P* = 0.242), and female gender (*P* = 0.235). Renal cell carcinoma showed a very strong association with the female gender and was removed (*P* < 0.001, Cramer’s V = 0.336), as the female gender had a lower univariate *P*-value. ICU admission showed a very strong association with vasopressors and was removed (*P* < 0.001, Cramer’s *V* = 0.738), as vasopressors had a lower univariate *P*-value. The capacity to endorse urinary symptoms showed a very strong association with vasopressors and was removed (*P* < 0.001, Cramer’s *V* = 0.478), as vasopressors had a lower *P*-value.

Table 4 shows the results for the binary logistic regression to further assess the relationship between fluconazole treatment duration and clinical treatment success. The Omnibus test (*P* = 0.02), Nagelkerke’s *R* squared (r^2^ = 0.381), and Hosmer-Lemeshow test (*P* = 0.562) indicated that the logistic regression had goodness-of-fit. Receipt of vasopressors was the only independent variable found to have a significant association with clinical treatment success on binary logistic regression and was found to be a negative predictor (*P* = 0.016; OR [95% CI]: 0.079 [0.015–0.626]). Fluconazole duration did not have a significant association with clinical treatment success on binary logistic regression (*P* = 0.503; OR 0.917 [95% CI: 0.712–1.181]).

**TABLE 4:** Binary logistic regression using urinary tract infection clinical treatment success as the dependent variable

	β	SE	Wald	dF	*P*-value	Or (95% CI)
Weight	−0.016	0.025	0.399	1	0.527	0.984 (0.936–1.034)
Serum creatinine	−0.501	0.327	2.351	1	0.125	0.606 (0.319–1.15)
Female gender	1.048	1.078	0.945	1	0.331	2.851 (0.345–23.584)
*C. tropicalis*	−1.801	1.226	2.156	1	0.412	0.165 (0.015–1.827)
Receipt of vasopressors	−2.535	1.054	5.780	1	0.016	0.079 (0.01–0.626)
Fluconazole loading dose	−0.881	1.111	0.628	1	0.428	0.414 (0.047–3.66)
Fluconazole duration	−0.087	0.129	0.449	1	0.503	0.917 (0.712–1.181)
Constant	6.78	2.969	5.215	1	0.022	880.091

## DISCUSSION

Current IDSA guidelines recommend 14 days of antifungal treatment for symptomatic *Candida spp*. UTI (6). However, this recommendation is based primarily on one study by Sobel and colleagues (7). They performed a prospective, multicenter, placebo-controlled study to evaluate the efficacy and safety of fluconazole 200 mg/day (*n* = 159) vs placebo (*n* = 157) for 14 days for candiduria in hospitalized patients who were asymptomatic or minimally symptomatic. However, minimally symptomatic represented <5% of the patients evaluated and was defined as afebrile with mild UTI symptoms that were not further defined. In the intent-to-treat analysis, candiduria cleared by day 14 in 79 (50%) and 46 (29%) of patients receiving fluconazole vs placebo, respectively (*P* < 0.0001). Despite the statistically significant difference, the candiduria clearance rates were relatively low in both cohorts, and the studied population was predominately asymptomatic, which was estimated to be approximately 95%. Therefore, it may be challenging to apply this study’s findings of 14 days of fluconazole to treatment duration recommendations for symptomatic *Candida spp*. UTI. To our knowledge, our study is the first to evaluate shorter vs longer treatment durations for symptomatic *Candida spp*. UTI.

In our study population, all patients had at least one symptom consistent with UTI, many had multiple symptoms, and most had imaging that also supported a UTI diagnosis. All patients who were included in this analysis (*n* = 103) were reviewed by an ID physician, either in ID consultation (60% in the 14-day cohort and 62.1% in the <14-day cohort) and/or antimicrobial stewardship, who supported the treatment of a *Candida spp*. UTI based on the patient history and/or information available. Furthermore, our study had strict exclusion criteria to avoid including patients with asymptomatic candiduria. In a 9-year period that screened 2,400 patients with a positive urine culture for *Candida spp*., only 103 patients met the study criteria for symptomatic UTI, which is consistent with real-world practice and the relatively low incidence of true symptomatic UTI compared to asymptomatic candiduria (2, 6, 7). All patients received fluconazole, and the median dose was 200 mg, which is consistent with that recommended by IDSA guidelines (6). Patients in each cohort were also comparable with regard to patient demographics, comorbidities, and baseline characteristics.

Clinical treatment success was greater than 90% in the patients treated with fluconazole for 14 days and <14 days for symptomatic *Candida spp*. UTI, suggesting both treatment durations were highly effective. Among patients who had a urinary catheter present, most were removed as supported by IDSA guidelines (6), which may have also contributed to the high clinical success rate. We also observed a non-significant difference in clinical treatment success with fluconazole for 14 days vs <14 days in symptomatic *Candida spp*. UTI, and the absolute between-group difference was less than 1%. Fluconazole treatment duration was, additionally, not found to be a significant predictor for clinical treatment success on binary logistic regression. These findings suggest that a longer fluconazole treatment duration, though highly effective, may not increase clinical treatment success vs the shorter duration in symptomatic *Candida spp*. UTI. ID physicians who used shorter treatment durations despite IDSA guideline recommendations for 14 days likely did so based on the overall data for shorter vs longer treatment durations for various infections (12), the patient’s clinical picture and treatment response, and their clinical judgment. Interestingly, the median treatment duration was 14 days in our 14-day cohort and 7 days in our <14-day cohort, suggesting 7 days of fluconazole may have comparable clinical treatment success vs 14 days in symptomatic *Candida spp*. UTI. This finding is consistent with study data for bacterial UTI including gram-negative bacteremia, where 7 days of treatment has generally become a “standard” treatment duration (8–12). Antifungal stewardship efforts are also supported by these data to use shorter vs longer treatment durations for fungal infections (13).

There were also no statistically significant differences in any of the secondary outcomes when using fluconazole for 14 days vs <14 days for symptomatic *Candida spp*. UTI. Hospital readmission due to *Candida spp*. UTI, microbiologic recurrence, and all-cause mortality had small (<5%), statistically non-significant differences between cohorts, and the 95% CI for their between-group differences was spread relatively evenly around zero. This suggests with some confidence that there may not have been a difference in hospital readmission due to *Candida spp*. UTI, microbiologic recurrence, and all-cause mortality in symptomatic *Candida spp*. UTI treated with fluconazole for 14 days vs <14 days. There was, however, a relatively large but statistically non-significant difference in hospital readmission from any cause, and the 95% CI for the between-group difference appeared to have a potential rightward skew. It is possible that our study may have been underpowered to detect this between-group difference, and there may be some possibility of a type II error. Hospital admission from any cause was, however, numerically lower in the <14-day cohort, and the 95% CI for the between-group difference suggests a potential, non-significant trend toward lower hospital readmission from any cause with fluconazole for <14 days.

This study is not without limitations. First, this study was retrospective in design, and we relied on progress notes for UTI symptom evaluation; however, these should be documented appropriately in the review of systems and/or physical examination sections if patients endorse or do not endorse such symptoms. Second, the definition used for clinical treatment success was in an effort to capture this outcome retrospectively since we were unable to survey patients following antifungal treatment and because limited definitions are available for retrospective evaluation of treatment durations on UTI outcomes (9). Third, this was performed at a single center with a relatively small sample size. Despite this, the total number of patients screened was 2,400, and we were able to apply strict inclusion and exclusion criteria to focus the evaluation solely on those patients with symptomatic *Candida spp*. UTI rather than asymptomatic candiduria, which is more commonly encountered in clinical practice (2, 7, 14). Although we recognize the possibility of random variation based on our sample size, we still believe our results are clinically meaningful, as there was a less than 1% absolute difference in clinical treatment success, and the 95% CI for the between-group difference appeared evenly distributed around zero. Similar observations were also made for the secondary outcomes of hospital readmission for *Candida spp*. UTI, microbiologic recurrence, and all-cause mortality. However, there was a large but statistically non-significant difference in the secondary outcome of hospital readmission from any cause, which was numerically lower in the <14-day cohort. Fourth, there were no patients included with *C. krusei* or fluconazole non-susceptible *C. glabrata*, and therefore, it is unclear if other antifungal treatment options for these *Candida spp*. would have similar findings. However, these *Candida spp*. are less common in clinical practice (2, 6, 7). Finally, we utilized a binary logistic regression to further assess the relationship between clinical treatment success and fluconazole treatment duration and did not observe any relationship between the two variables. Any further results within the logistic regression should be interpreted with caution, as there was some potential for overfitting. With that said, the results from our binary logistic regression were not surprising, as all non-significant variables were also non-significant in univariate analysis, while the lone significant variable was also significant in univariate analysis. Additionally, the lone significant variable on univariate analysis and binary logistic regression was the receipt of vasopressors, which had an inverse relationship with clinical treatment success.

### Conclusions

Among patients who received shorter (median 7 days) compared to IDSA guideline recommended longer (14 days) treatment durations of fluconazole for symptomatic *Candida spp*. UTI, no statistically significant differences in clinical treatment success, hospital readmission, hospital readmission due to *Candida spp*. UTI, microbiologic recurrence, or all-cause mortality were identified. While additional data are needed to further evaluate fluconazole treatment durations for *Candida spp*. UTI, these data suggest that shorter fluconazole treatment durations may have similar clinical effectiveness vs longer durations in symptomatic *Candida spp*. UTI.
